# Highly Pathogenic Avian Influenza Clade 2.3.4.4 Subtype H5N6 Viruses Isolated from Wild Whooper Swans, Mongolia, 2020

**DOI:** 10.3201/eid2704.203859

**Published:** 2021-04

**Authors:** Sol Jeong, Nyamsuren Otgontogtokh, Dong-Hun Lee, Bayarmagnai Davganyam, Sun-Hak Lee, Andrew Y. Cho, Erdene-Ochir Tseren-Ochir, Chang-Seon Song

**Affiliations:** Konkuk University, Seoul, South Korea (S. Jeong, S.-H. Lee, A.Y. Cho, C.-S. Song);; Mongolian University of Life Sciences, Ulaanbaatar, Mongolia (N. Otgontogtokh, B. Davganyam, E.-O. Tseren-Ochir);; University of Connecticut, Storrs, Connecticut, USA (D.-H. Lee)

**Keywords:** influenza, zoonoses, viruses, Mongolia, highly pathogenic avian influenza, wild whooper swans, H5N6, respiratory infections

## Abstract

We identified clade 2.3.4.4 highly pathogenic avian influenza A(H5N6) viruses from whooper swans (*Cygnus cygnus*) found dead in Mongolia. The identification of these infections in wild birds in this area is of concern because of the potential for virus dissemination during fall migration.

Highly pathogenic avian influenza (HPAI) H5Nx viruses have been continuous threat to poultry and public health since the detection of A/Goose/Guangdong/1/1996(H5N1) (Gs/GD) in 1996 in Guangdong Province, China. The Gs/GD-lineage has evolved into 10 genetically distinct clades (0–9) and subclades (*1*). A novel clade 2.3.4.4 of H5Nx viruses bearing multiple neuraminidase subtypes, including N2, N5, N6, and N8, has been identified in China since 2008 (*2*), and the H5 genes have been phylogenetically differentiated into 4 subgroups (A–D) (*3*). Clade 2.3.4.4 H5N6 viruses have been causing worldwide epizootics in poultry and wild birds, and human cases have also been reported since 2014 (*4*). In this study, we report the identification and genetic analysis of 2 HPAI clade 2.3.4.4 H5N6 viruses isolated from whooper swan carcasses in central Mongolia during April 2020.

Wild whooper swan (*Cygnus cygnus*) carcasses were found in April 2020 on the banks of 2 small ponds (48°25′39.8′′N, 102°36′09.6′′E, and 47°56′22.0′′N, 102°32′54.0′′E) around the Orkhon River located nearby Khunt Lake (Bulgan Province, 48°25′59.8′′N, 102°34′51.1′′E) and Doitiin Tsgaaan Lake (Arkhangal Province, 47°34′20.1′′N, 102°31′45.4′′E) (Appendix 1 Figure 1). These lakes are a major stopover site of migratory wild birds in central Mongolia between their breeding sites in the north and the wintering sites in the south and are also major breeding and molting habitats of wild bird species, including whooper swan. The lakes were the outbreak sites of HPAI H5N1 in wild birds in 2005, 2006, and 2009 (*5*). Two HPAI viruses, A/Whooper swan/Mongolia/24/2020(H5N6) and A/Whooper swan/Mongolia/25/2020(H5N6), referred to as MN-H5N6/2020 viruses in this article, were isolated from 2 brain tissue samples of whooper swan carcasses. We conducted whole-genome sequencing (*6*) and phylogenetic analysis on the isolates (Appendix 1). The nucleotide sequences have been deposited in GenBank (accession nos. MT872354–69).

The 2 MN-H5N6/2020 viruses shared high nucleotide similarity (99.65%–100%) across all 8 gene segments. Polybasic amino acid motif on the cleavage site of hemagglutinin (HA) genes (PLRERRRKR/G) suggested that the MN-H5N6/2020 viruses are HPAI. BLAST (https://blast.ncbi.nlm.nih.gov/Blast.cgi) and GISAID (https://platform.gisaid.org) searches showed that the MN-H5N6/2020 viruses share high nucleotide identity (99.4%–99.9%) across all 8 gene segments with the HPAI H5N6 viruses, referred to as Xinjiang-H5N6/2020 viruses in this article, isolated from wild swans (mute swans [*Cygnus olor*] and whooper swans) during January 2020 in Xinjiang Province, China (Table 1) (*7*), which is located ≈4,800 km southwest of the isolation sites of the MN-H5N6/2020 viruses. These results suggested that the MN-H5N6/2020 viruses might have been introduced through the Central Asian flyway to central Mongolia most likely during early spring migration in 2020.

**Table 1:** Nucleotide sequence identities between each gene segment of MN-H5N6/2020 highly pathogenic avian influenza virus isolates from Mongolia, 2020, and the isolates with the highest homology in the GISAID and GenBank databases*

Gene	Accession no.	Virus	% Identity
PB2	EPI1718955	A/Whooper swan/Xinjiang/3/2020(H5N6)	99.74–99.83
PB1	EPI1718956	A/Whooper swan/Xinjiang/3/2020(H5N6)	99.44
PA	EPI1719034	A/Whooper swan/Xinjiang/13/2020(H5N6)	99.78
HA	EPI1718990	A/Whooper swan/Xinjiang/7/2020(H5N6)	99.60–99.66
NP	EPI1718951	A/Whooper swan/Xinjiang/3/2020(H5N6)	99.74–99.81
NA	EPI1719037	A/Whooper swan/Xinjiang/13/2020(H5N6)	99.65–99.72
MP	EPI1719033	A/Whooper swan/Xinjiang/13/2020(H5N6)	99.70
NS	EPI1719032	A/Whooper swan/Xinjiang/13/2020(H5N6)	99.54

*As of 2020 Jul 16. GISAID, https://platform.gisaid.org. HA, hemagglutinin; MP, matrix protein; NP, nucleoprotein; NS, nonstructual protein PA, acidic polymerase; PB1, basic polymerase 1; PB2, basic polymerase 2.

In the maximum-likelihood phylogenetic trees, all 8 gene segments of the MN-H5N6/2020 were closely clustered with the sequences of the Xinjiang-H5N6/2020 viruses and the H5N6 viruses of clade 2.3.4.4 group C isolated during 2016–2019 in China, Vietnam, and Russia, including human isolates (Appendix 1 Figure 2). The phylogenetic relationship and high nucleotide identity indicated that the MN-H5N6/2020 viruses possess the identical genome constellation with the Xinjiang-H5N6/2020 viruses (*7*). The time of most recent common ancestor for each gene of the MN-H5N6/2020 viruses and the Xinjiang-H5N6/2020 viruses ranged from May to December 2019, suggesting that the MN-H5N6/2020 viruses and the Xinjiang-H5N6/2020 viruses had diverged from a common ancestor most likely during the second half of the previous year (Table 2; Appendix 1 Figure 3). The time of most recent common ancestor for each gene of the MN-H5N6/2020 viruses ranged from January through March 2020. These data and understanding of waterfowl migration patterns suggest that H5N6 viruses were maintained among wild birds during fall and winter 2019 and reached Mongolia by late winter, most likely carried by long-distance flights of infected migrating wild birds during spring migration. A previous satellite-tracking study of whooper swans between northern China and Mongolia showed that the most stable period for the wintering population of whooper swans in the Sanmenxia Reservoir area was from late December to early January (*8*). For spring migration, departure dates of the wintering population of whooper swans ranged from February 17 to March 27, and arrival dates at the breeding sites in Mongolia ranged from February 27 to May 23. This spring bird migration pattern coincided with the timing and direction of H5N6 virus transmission between Xinjiang and Mongolia.

**Table 2:** tMRCA of each gene segment of H5N6 highly pathogenic avian influenza viruses from Mongolia, 2020*

Gene	tMRCA† of MN-H5N6/2020 viruses			tMRCA of MN-H5N6/2020 and Xinjiang-H5N6/2020 viruses	
	Mean	95% HPD‡ range		Mean	95% HPD range
PB2	Feb 2020	Jan–Mar 2020		Dec 2019	Dec 2019
PB1	Feb 2020	Jan–Mar 2020		May 2019	Dec 2018–Aug 2019
PA	Jan 2020	Dec 2019–Mar 2020		Sep 2019	Jun–Nov 2019
HA	Jan 2020	Oct 2019–Feb 2020		Jul 2019	Mar–Oct 2019
NP	Jan 2020	Nov 2019–Mar 2020		Nov 2019	Aug–Dec 2019
NA	Feb 2020	Dec 2019–Mar 2020		Jul 2019	Jan–Oct 2019
MP	Mar 2020	Jan–Mar 2020		Jul 2019	Feb–Oct 2019
NS	Feb 2020	Dec 2019–Mar 2020		Nov 2019	Sep–Dec 2019

*HA, hemagglutinin; HPD, highest posterior density; MP, matrix protein; NP, nucleoprotein; NS, nonstructural protein PA, acidic polymerase; PB1, basic polymerase 1; PB2, basic polymerase 2; tMRCA, time to the most recent common ancestor. †tMRCA estimated by using Bayesian molecular clock analysis. It represents the potential existing timing of a common ancestral node.

The MN-H5N6/2020 and the Xinjiang-H5N6/2020 viruses had mutations associated with increased HA receptor binding affinity to human-like receptor (α-2,6 sialic acid), including D94N, S133A, S154N, and T156A (H5 numbering) (*9*) (Appendix 2 Table 2), although they maintained the amino acids related to the binding tropism to avian-like (α-2,3 sialic acid) receptors (222Q and 224G). Unlike 7 H5N6 human isolates of clade 2.3.4.4 group C, the MN-H5N6/2020 and the Xinjiang-H5N6/2020 viruses had amino acid substitution at HA position 188 (H5 numbering), from threonin to isoleucine, which is known to enhance receptor binding affinity to human-like receptor (*10*). In the maximum-likelihood phylogenetic tree and maximum clade credibility tree of polymerase basic 2 gene, the closest isolates of the MN-H5N6/2020 and Xinjiang-H5N6/2020 viruses were human H5N6 isolates from China (Table 2; Appendix 1 Figure 2, 3).

Wild migratory birds have played an important role in disseminating and maintaining of Gs/GD lineage HPAI H5Nx viruses, as observed in the epizootics of H5N1 clade 2.2 during 2005–2006 (*11*), H5N1 clade 2.3.2 in 2009 (*12*), clade 2.3.2.1c in 2015 (*13*), and clade 2.3.4.4 since 2014 (*14*). During widespread dissemination of the HPAIV clade 2.2 during 2005–2006 and clade 2.3.2 in 2009, these viruses were also detected from wild birds at Doitiin Tsgaaan Lake and Khunt Lake, highlighting that these areas are useful locations for monitoring of HPAI in wild birds as a pathway for the spread of HPAI during migration of waterfowl. Identifying 2 H5N6 HPAI viruses in wild waterfowl in this area signifies the potential for wide spread of this clade 2.3.4.4 H5N6 viruses during the 2020 fall migration.

Since the first report of a human infection with HPAI clade 2.3.4.4 H5N6 virus in Sichuan Province, China, in April 2014 (*4*), a total of 24 cases had been reported from China as of August 2020 (*15*). The mammalian host-specific markers found in the MN-H5N6/2020 viruses suggest that these viruses are potentially infectious for mammals. Considering the possibility of future dispersal of the H5N6 HPAI viruses through wild birds and the presence of mammalian host-specific genetic markers, enhanced active surveillance in wild birds, poultry, and mammals is needed to monitor spread and understand the potential for zoonotic infection.

Appendix 1 Additional information about highly pathogenic avian influenza clade 2.3.4.4 subtype H5N6 viruses isolated from wild whooper swans, Mongolia, 2020.

Appendix 2 GISAID data for highly pathogenic avian influenza clade 2.3.4.4 subtype H5N6 viruses isolated from wild whooper swans, Mongolia, 2020.
