# Supplementary material for: Highly Pathogenic Avian Influenza Clade 2.3.4.4 Subtype H5N6 Viruses Isolated from Wild Whooper Swans, Mongolia, 2020
Source: Emerg Infect Dis. 2021 Apr;27(4):1181–3. doi: 10.3201/eid2704.203859 (PMC8007304; doi:10.3201/eid2704.203859)
Supplement: Appendix 1 — Additional information about highly pathogenic avian influenza clade 2.3.4.4 subtype H5N6 viruses isolated from wild whooper swans, Mongolia, 2020. [file 20-3859-Techapp-s1.pdf]

Article DOI: https://doi.org/10.3201/eid2704.203859

Highly Pathogenic Avian Influenza Clade 2.3.4.4 Subtype H5N6 Viruses Isolated from Wild Whooper Swans, Mongolia, 2020

Appendix 1

Methods

Virus Isolation

For avian influenza virus (AIV) screening, viral RNA was extracted from brain samples using taco Nucleic Acid Automatic Extraction System (GeneReach Biotechnology Corp., Taiwan) and taco DNA/RNA Extraction Kit (GeneReach Biotechnology Corp., Taiwan) and screened for AIV by insulated isothermal PCR (iiPCR) using POCKIT Micro Duo Nucleic Acid Analyzer (GeneReach Biotechnology Corp., Taiwan) with Influenza A Virus Detection kit (GeneReach Biotechnology Corp., Taiwan) according to the manufacturer's instructions. AIV screening result was confirmed by real-time reverse transcription-PCR (rRT-PCR) (*1*). Supernatant of tissue homogenates was inoculated into 9–11-day old chicken embryonated eggs in Biosafety Level 3 (BSL-3) facilities at State Central Veterinary Laboratory of Mongolia for virus propagation. Allantoic fluids were harvested after 72 h incubation and tested for hamagglutinin (HA) activity using 10% chicken red blood cells.

Whole-Genome Sequencing

RNA was extracted from HA-positive allantoic fluid using TRIzol Reagent (Thermo Fisher Scientific, MA, USA) and RNeasy Mini Kit (QIAGEN, CA, USA) according to the manufacturer's instructions with slight modifications. Briefly, 200 µl of allantoic fluid was treated with 200 µl of TRIzol Reagent and incubated for 5 min at room temperature. Then, 100 µl of chloroform was added and incubated for 2 min at room temperature. After centrifuging the mixture at 13,000 rpm for 15 minuates at 4°C, the RNA and water phase was mixed with 100% ethanol (1:1v). After transferring the mixture to the spin column of RNeasy Mini kit, RNA was extracted following the manufacturer's instructions.

The full-length genome sequencing was performed using the Ion Torrent Personal Genome Machine (Life Technologies, CA, USA) according to the manufacturer's instructions as previously described (*2*). De novo and directed assembly were carried out using the Geneious assembler and mapper in Geneious R9 (http://www.geneious.com) using default parameters.

Phylogenetic Analysis

The sequences of the highly pathogenic avian influenza (HPAI) viruses representing each subgroups of H5 clade 2.3.4.4 and low pathogenic avian influenza viruses were retrieved from GenBank (https://www.ncbi.nlm.nih.gov/genomes/FLU) and GISAID Epiflu (https://platform.gisaid.org) databases for comparative phylogenetic analysis. Complete coding regions were aligned using MAFFT (https://mafft.cbrc.jp/alignment/software/). The maximum-likelihood (ML) tree of each gene was estimated using RAxML (*3*) using general time-reversible (GTR) nucleotide substitution model and discrete gamma distribution with 1,000 rapid bootstrap replicates. To estimate the time of most recent common ancestry (tMRCA) for each gene segment, we performed Bayesian relaxed clock phylogenetic analysis using BEAST v1.8.4. We applied the Hasegawa–Kishino–Yano nucleotide substitution model, and the Gaussian Markov random field (GMRF) Bayesian skyline coalescent tree prior (*4*). A Markov Chain Monte Carlo method to sample trees and evolutionary parameters was run for 50 million generations. Three independent runs were combined for analyses to ensure that an adequate effective sample size (200) was reached for relevant parameters. BEAST output was analyzed with TRACER v1.6 (https://beast.bio.ed.ac.uk/tracer) with 10% burn-in. A maximum clade credibility tree was generated for each dataset by using TreeAnnotator v1.8.4 and visualized with FigTree v1.4.2 (https://tree.bio.ed.ac.uk).

References

1. Spackman E, Senne DA, Bulaga LL, Myers TJ, Perdue ML, Garber LP, et al. Development of real-time RT-PCR for the detection of avian influenza virus. Avian Dis. 2003;47(Suppl):1079–82. PubMed https://doi.org/10.1637/0005-2086-47.s3.1079

2. Kwon JH, Jeong S, Lee DH, Swayne DE, Kim YJ, Lee SH, et al. New reassortant clade 2.3.4.4b avian influenza A(H5N6) virus in wild birds, South Korea, 2017–18. Emerg Infect Dis. 2018;24:1953–5. PubMed https://doi.org/10.3201/eid2410.180461

3. Stamatakis A. RAxML version 8: a tool for phylogenetic analysis and post-analysis of large phylogenies. Bioinformatics. 2014;30:1312–3. PubMed https://doi.org/10.1093/bioinformatics/btu033

4. Suchard MA, Weiss RE, Sinsheimer JS. Bayesian selection of continuous-time Markov chain evolutionary models. Mol Biol Evol. 2001;18:1001–13. PubMed https://doi.org/10.1093/oxfordjournals.molbev.a003872

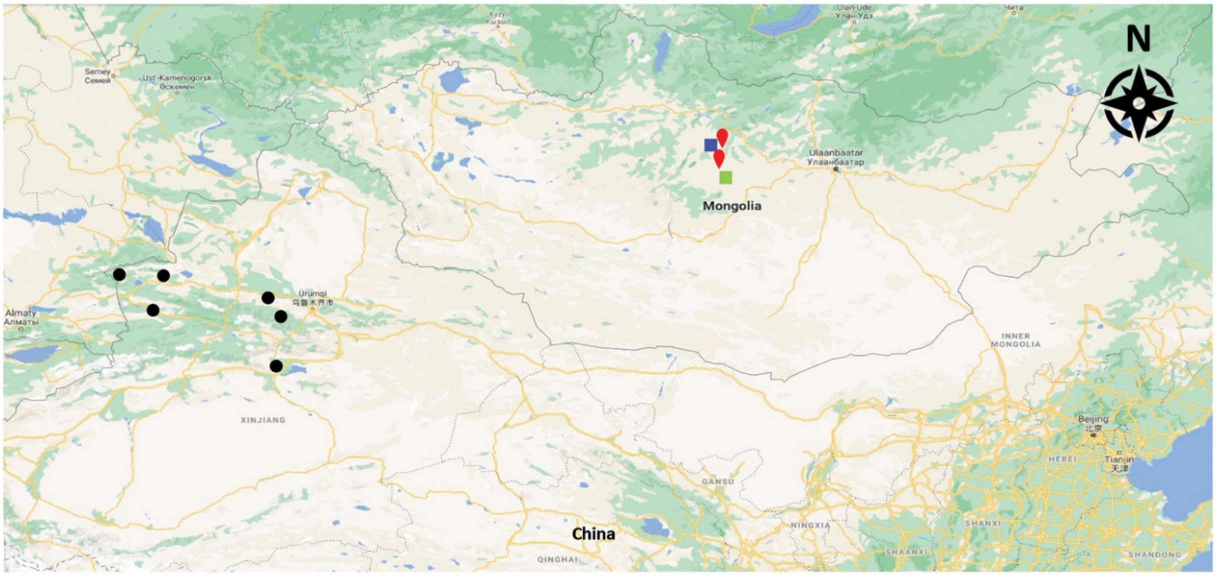

Appendix Figure 1. Sampling locations of MN-H5N6/2020 (indicated with red circle) and Xinjiang-H5N6/2020 viruses (indicated with black circle). Lakes located nearby the sampling locations of MN-H5N6/2020 viruses are indicated with squares (Khunt Lake, blue; Doitiin tsgaaan Lake, green). Map was retrieved from Google Maps (Google. google.com/maps/. October 29th, 2020).

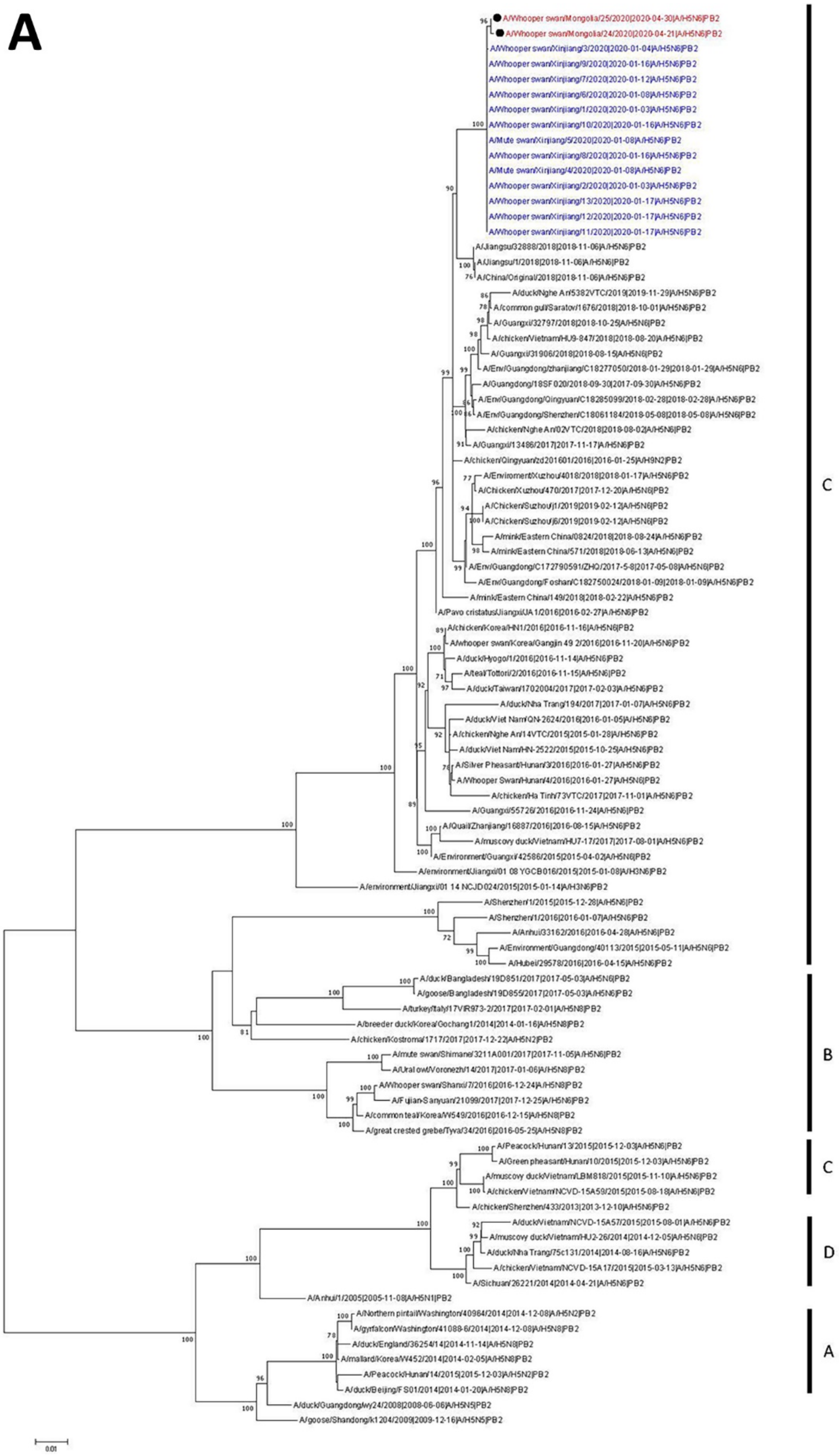

A
C
B
C
D
A
0.01

B

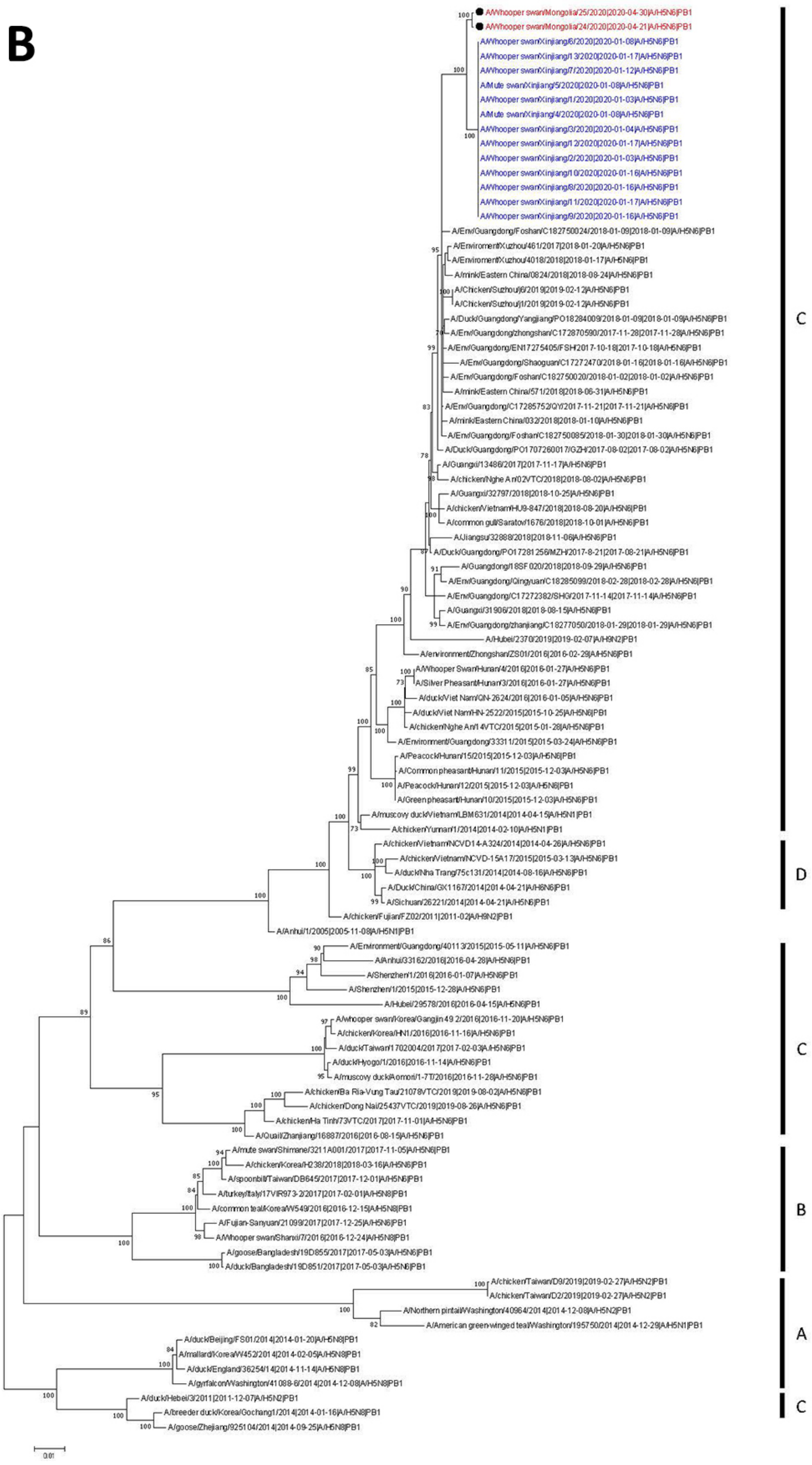

C

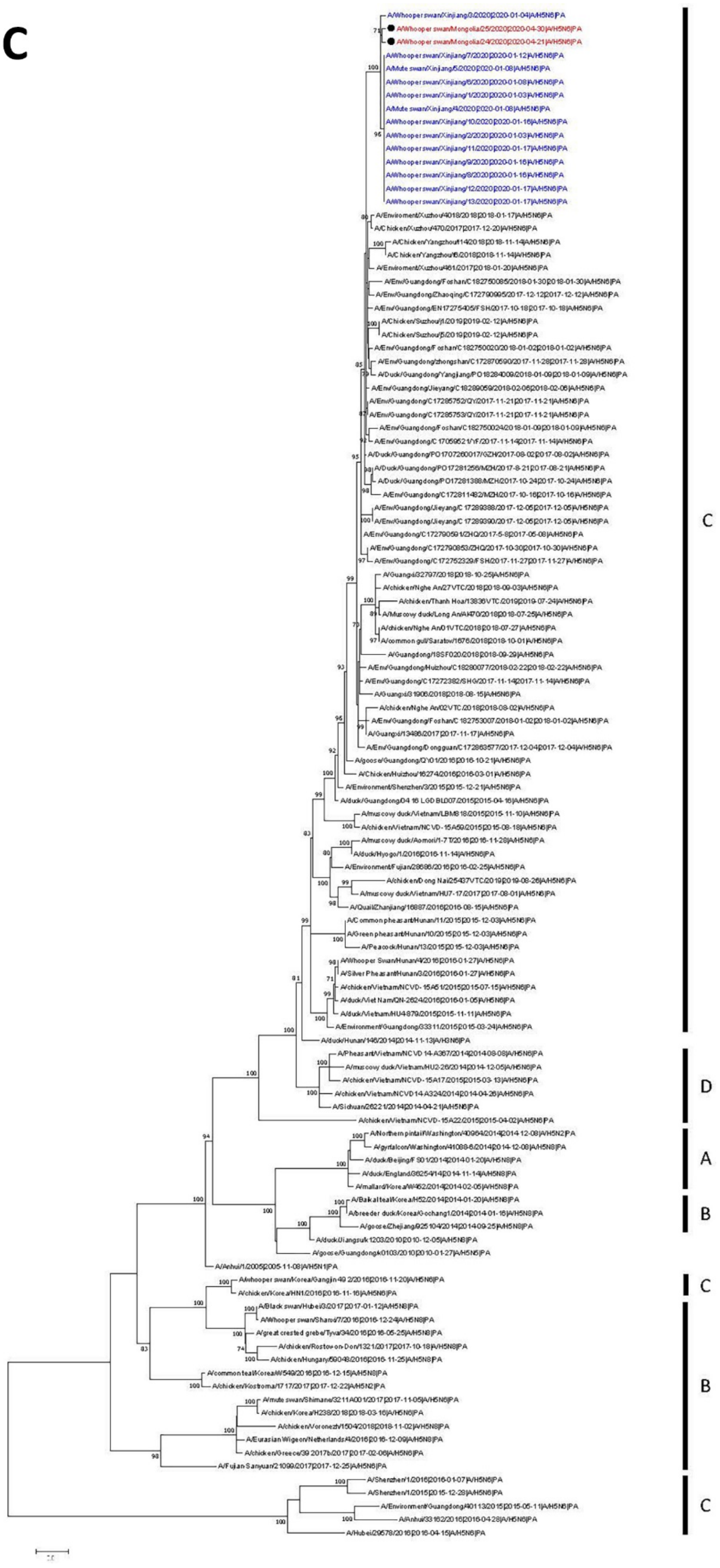

D

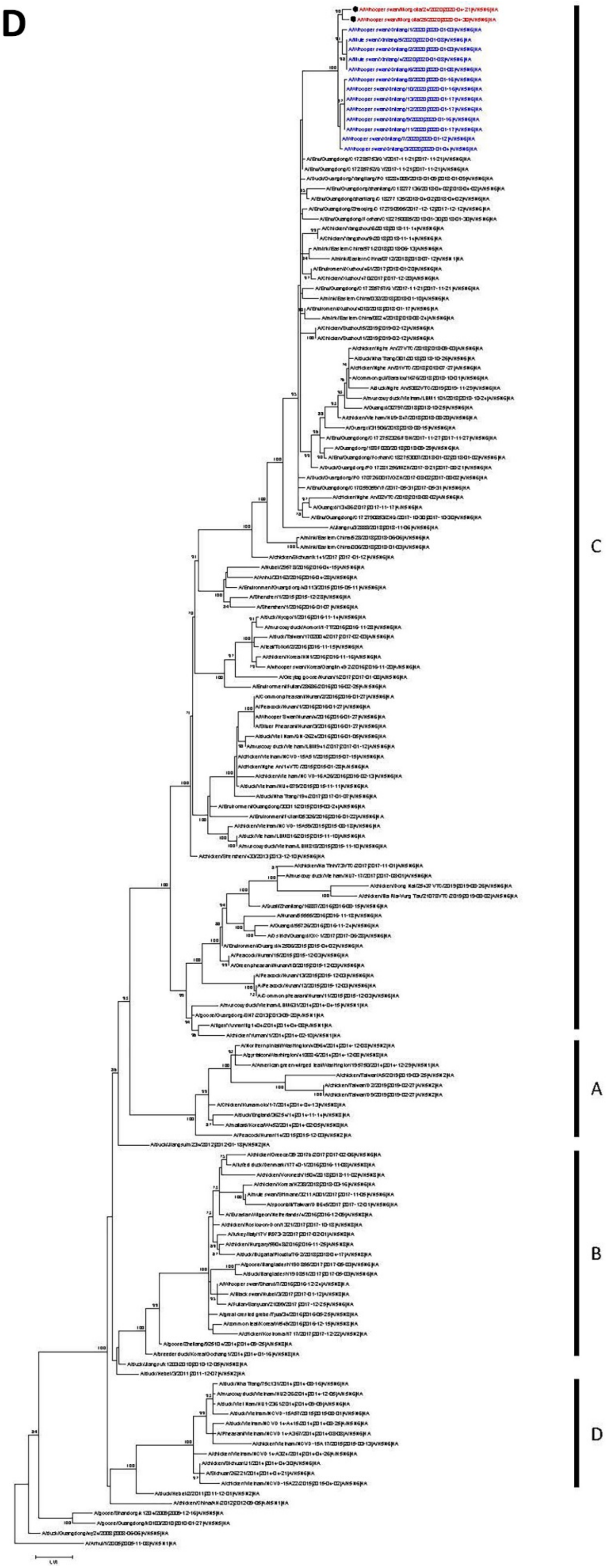

E

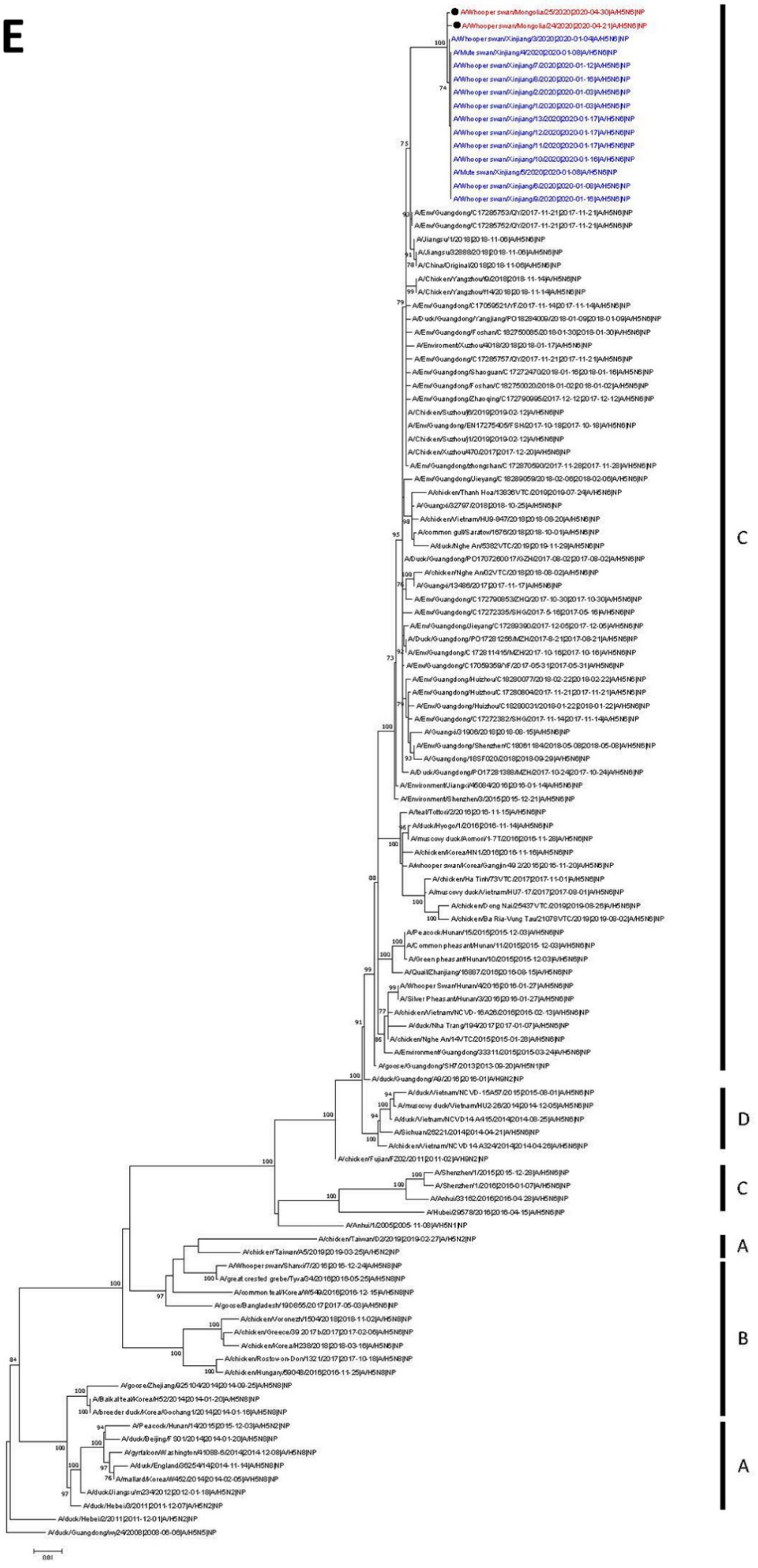

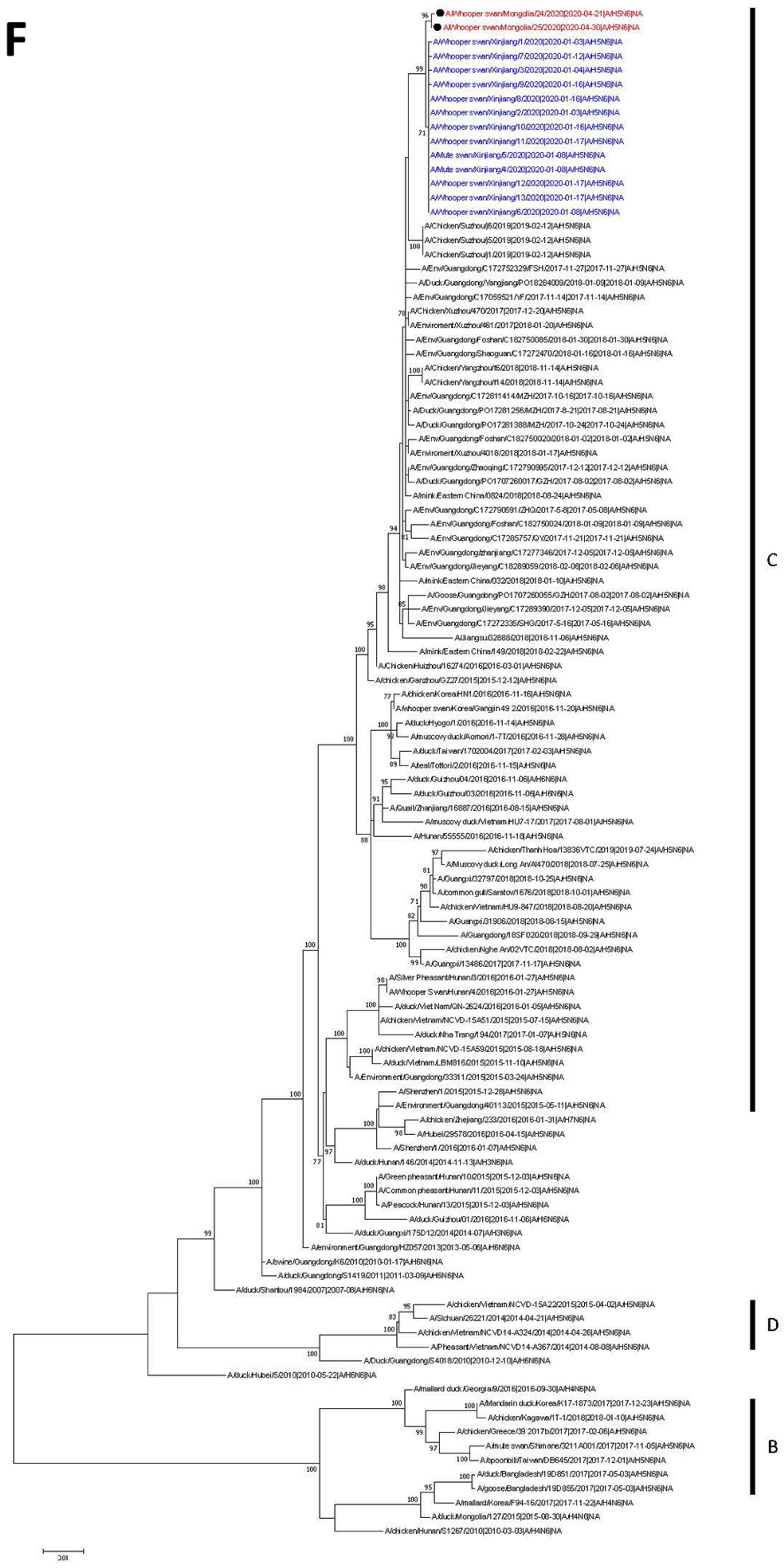
F
C
D
B

G

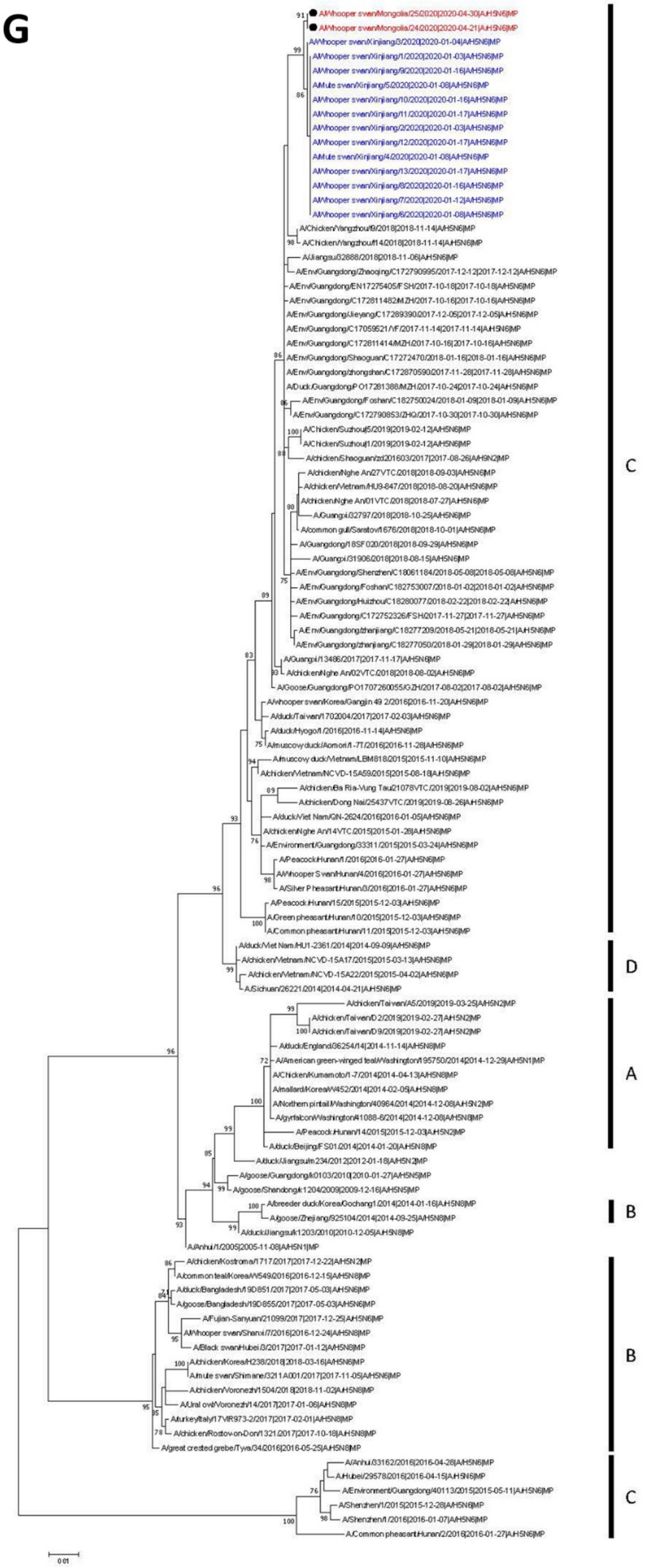

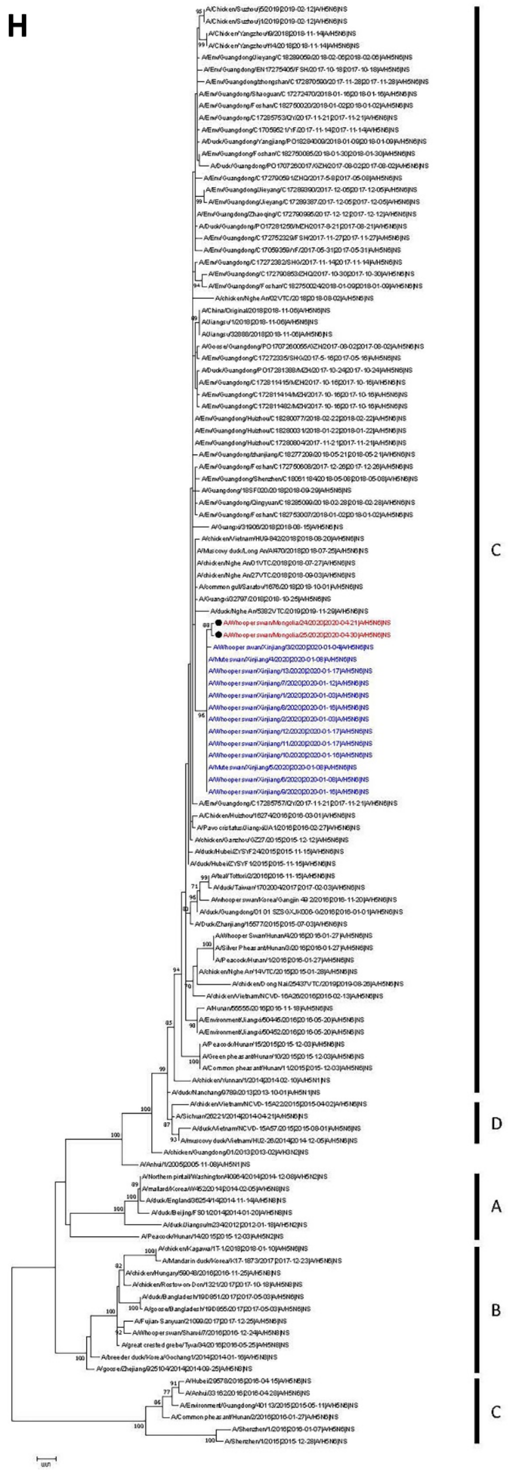

Appendix Figure 2. Maximum-likelihood phylogenetic trees of eight gene segments. Bootstrap values over 70% are shown. Taxa was colored according to the isolation location (red, Mongolia; blue, Xinjiang, China). A, polymerase basic 2 (PB2); B, polymerase basic 1 (PB1); C, polymerase acidic (PA); D, haemagglutinin (HA); E, nucleoprotein (NP); F, neuraminidase (NA); G, matrix (M); H, nonstructual (NS).

A

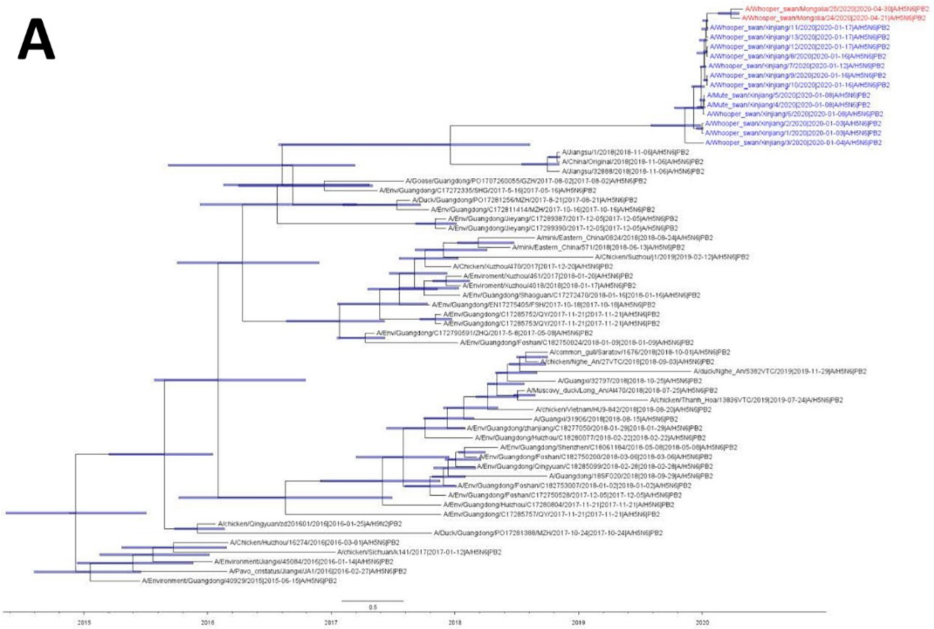

B

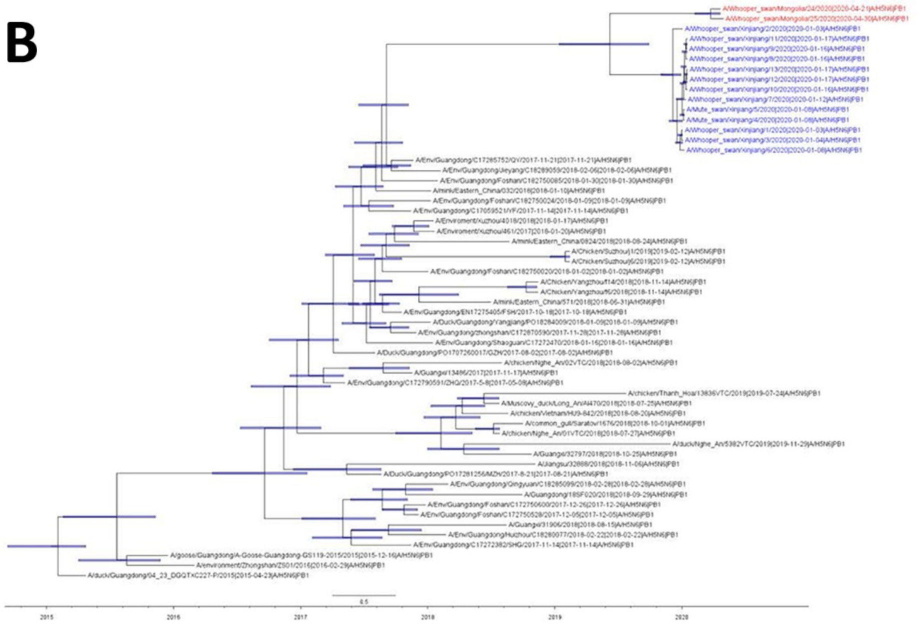

C

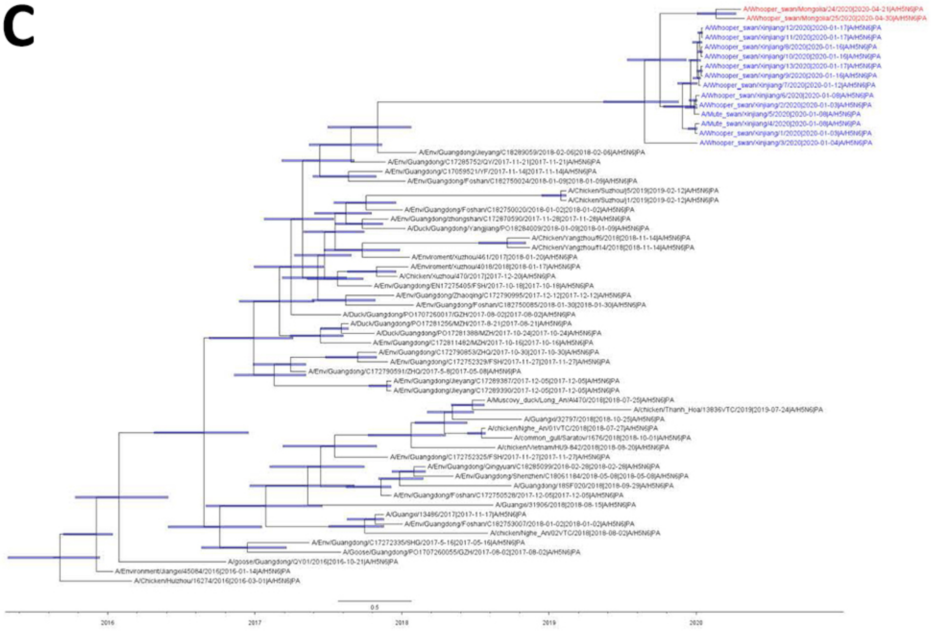

D

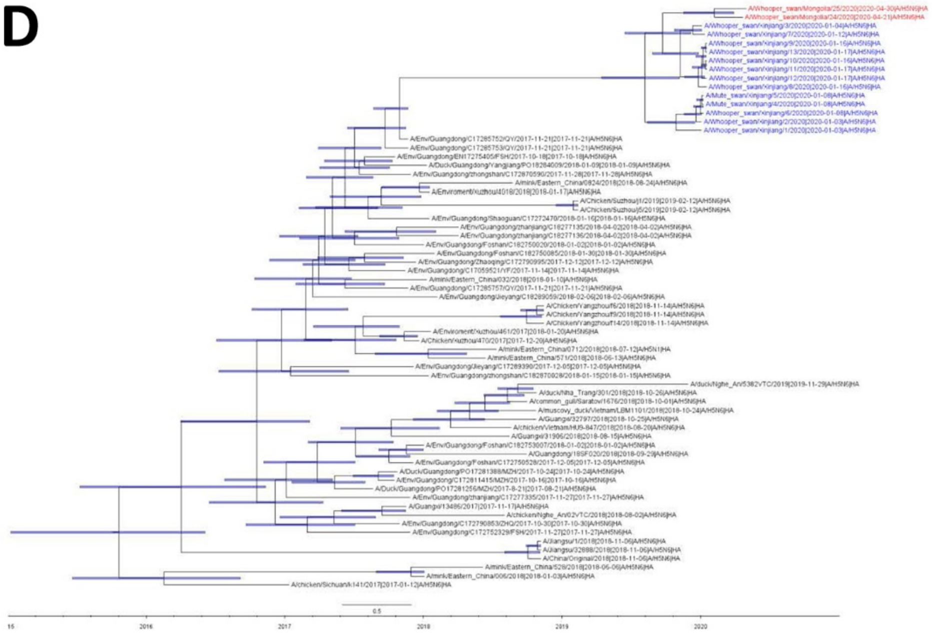

E

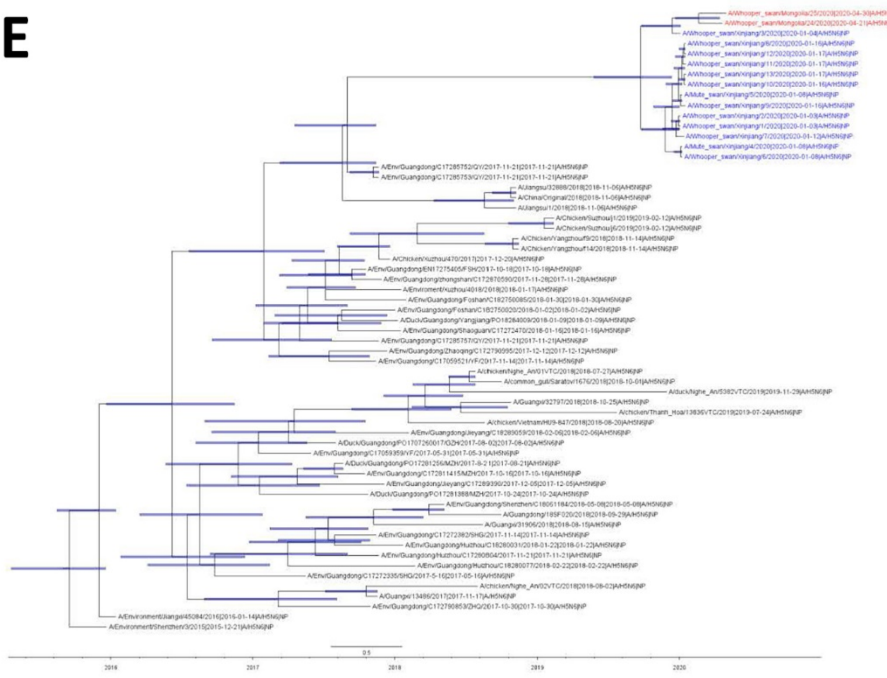

F

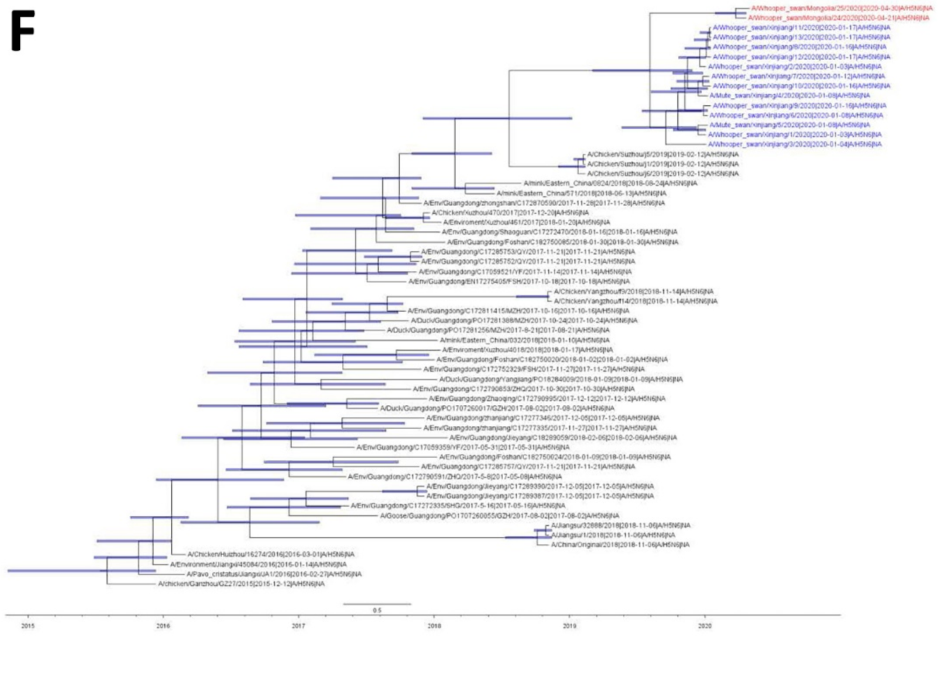

G

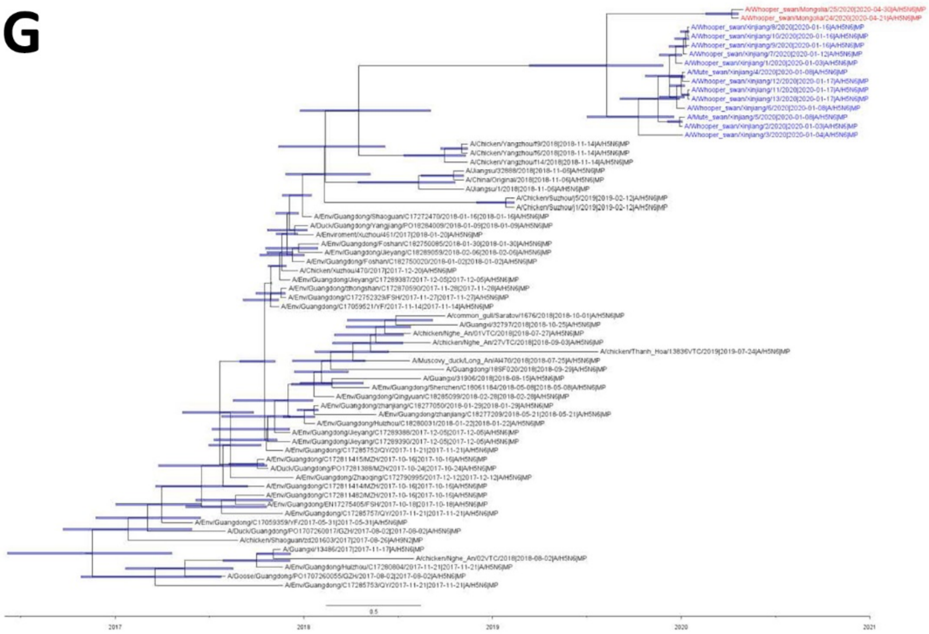

H

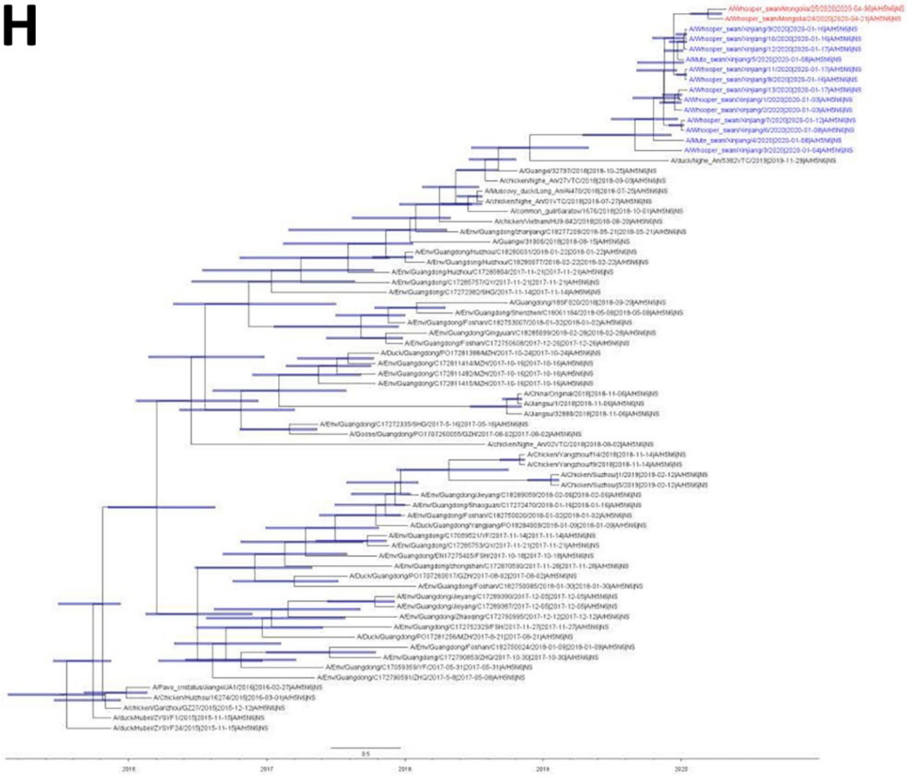

Appendix Figure 3. Temporally structured maximum clade credibility phylogenetic trees of eight gene segments of clade 2.3.4.4. Time scale is shown on the horizontal axis. Taxa was colored according to the

isolation location (red, Mongolia; blue, Xinjiang, China). The horizontal bars on each branch indicate the 95% highest posterior density (HPD) intervals of the most common ancestor. A, polymerase basic 2 (PB2); B, polymerase basic 1 (PB1); C, polymerase acidic (PA); D, haemagglutinin (HA); E, nucleoprotein (NP); F, neuraminidase (NA); G, matrix (M); H, nonstructual (NS).
